# Barriers and facilitators to managing medicines at home post-myocardial infarction: a qualitative systematic review

**DOI:** 10.1007/s11096-025-01927-x

**Published:** 2025-06-04

**Authors:** Fatma El-Komy, Laura J. Sahm, Stephen Byrne, Margaret Bermingham, Michelle O’Driscoll

**Affiliations:** 1 Pharmaceutical Care Research Group, School of Pharmacy, University College Cork, Cork, T12 YT20 Ireland; 2 Pharmacy Department, Mercy University Hospital, Grenville Place, Cork, Ireland

**Keywords:** Adherence, Caregivers, Medication, Medication management, Myocardial infarction, Patients

## Abstract

**Background:**

Over three million people annually experience myocardial infarction (MI). As MI survival rates increase, so does the importance of secondary prevention medications. International guidelines recommend using several medications to prevent further morbidity.

**Aim:**

To synthesise the qualitative literature on the facilitators and barriers faced by MI survivors and their families/caregivers regarding medication management and, thus, medication adherence post-discharge.

**Method:**

This systematic review was conducted and reported following the PRISMA-2020 guidelines. Five databases were searched from inception until the 13th of August 2024. The inclusion criteria were articles focused on people aged 18 years or older who experienced MI and were discharged from acute care settings to home settings, as well as caregivers of individuals who met the above-mentioned criteria. Qualitative and mixed-methods studies with qualitative elements were deemed eligible for inclusion. The theoretical domain framework was used to analyse the findings. The quality of the included studies was assessed using the JBI Critical Appraisal tool for qualitative research. The Confidence in the Evidence from the reviews of qualitative research approach was applied to assess confidence in qualitative evidence synthesis.

**Results:**

Of the 14,002 titles, 11,354 remained after duplicates were removed. Of the 234 full-text screenings, fifteen were included. A total of 533 people who experienced MI and 25 spouses from eight different countries were included. The most prominent facilitator that emerged was “beliefs about consequences”, whilst “lack of knowledge” and “environmental context and resources” were the most prominent barriers to medication management reported.

**Conclusion:**

Patients face multiple challenges that affect their medication adherence post-MI. These findings highlight important considerations for creating an individualised, tailored approach to enhance medication adherence post-MI.

Systematic review registration: PROSPERO CRD42023424844.

**Supplementary Information:**

The online version contains supplementary material available at 10.1007/s11096-025-01927-x.

## Impact statements

- This qualitative systematic review shows that managing medicines at home post-myocardial infarction is a complex process influenced by patient beliefs, healthcare interactions, and social support, requiring a more personalised approach to adherence interventions.
- Providing accessible and timely information about secondary prevention medicines can positively influence medication adherence.
- Caregiver and family support can influence medication adherence both positively and negatively. Additionally, a lack of knowledge about medications can hinder adherence.
- Healthcare interventions should move beyond general adherence strategies and incorporate tailored, patient-centred approaches that address both practical and psychological challenges post-myocardial infarction.

## Introduction

According to the World Health Organisation (WHO), in 2019, around 17.9 million people died from cardiovascular diseases (CVDs) worldwide; most of these deaths are due to stroke or Myocardial Infarction (MI) [1]. Acute coronary syndrome (ACS) comprises a range of conditions involving (i) recent changes in clinical symptoms due to sudden reductions in blood flow to the heart, typically caused by plaque rupture and thrombus formation, leading to ischemia and myocardial damage, (ii) changes on electrocardiogram (ECG), and /or (iii) elevations in cardiac troponin. These conditions are ST-Elevation Myocardial Infarction (STEMI), Non-ST-Elevation Myocardial Infarction (NSTEMI), and Unstable Angina (UA). STEMI and NSTEMI are both types of MI [2]. Patients may be diagnosed with acute myocardial infarction (AMI) based on cardiac troponin elevation [3]. The survival rate of ACS is contingent upon variables such as the severity of the disease, the timeliness of medical attention, and the patient's general well-being [4]. Recent studies indicate that the overall survival rate for ACS has increased over time, mainly due to advancements in medical technology, pharmaceuticals, and public awareness programs that promote heart health and encourage early intervention [3]. MI is one of the most life-threatening coronary events. Internationally, over three million people annually experience STEMI [5], with significantly more experiencing non-STEMI. The recently released 2023 CVD prevention guidelines from the European Society of Cardiology (ESC) aim to assist healthcare providers in identifying the most effective approaches to treating modifiable risk factors in patients with a history of atherosclerotic cardiovascular disease (ASCVD) [3, 6]. Following ACS, patients are prescribed aspirin/purinergic receptor P2Y, G-protein coupled, 12 protein (P2Y12) inhibitors as antiplatelet therapy, beta-blockers, mineralocorticoid Receptor Antagonists (MRA), statins, and angiotensin-converting-enzyme inhibitors (ACEIs) or angiotensin receptor blockers (ARBs) as this combination has a demonstrable impact on reducing CVD morbidity and mortality in individuals [3, 7].

Secondary prevention aims to protect the patient from further cardiovascular events through lifestyle modifications and cardiac rehabilitation [8]. However, its efficacy is compromised when there is a high prevalence of medication non-adherence or cessation of therapy altogether [8]. Medication non-adherence has been defined as a situation where an individual's behaviour does not align with the prescribed treatment recommendations from their healthcare provider. This can present as intentional non-adherence, e.g. a person decides not to follow the treatment regimen, or unintentional, e.g. a person forgets to take their medicines [9, 10]. Measurement of adherence does not have a gold standard, and some studies may use subjective self-report or define it as consuming at least 80% of the prescribed doses, whilst others may rely on measures such as the Medication Events Monitoring Systems (MEMS) [9–12]. Adherence is considered to be a cornerstone of MI secondary prevention, with numerous clinical trials demonstrating the efficacy of evidence-based treatments in reducing cardiovascular events [8, 13]. However, adherence is not a definitive measure of improved outcomes, as studies have shown that even in placebo groups, higher adherence is associated with better survival, likely reflecting unmeasured healthy behaviours occurring simultaneously [14, 15] Nonetheless, non-adherence to medications post-MI is linked to a heightened risk of experiencing negative health outcomes, adverse clinical events, and even death [9].

Factors contributing to non-adherence with medications post-MI are diverse and may encompass financial constraints, limited accessibility, adverse effects, misconceptions regarding potential advantages and disadvantages, and other related issues [16, 17]. Medication non-adherence is widespread, but it has a particularly significant impact on patients in resource-limited environments. The rates of adherence to long-term medication in low-income countries can be as low as 10% among patients with prior CVD [9]. With the increasing prevalence of ASCVD worldwide, non-adherence will likely emerge as a significant factor contributing to poor health outcomes [18]. The WHO's 'Global Plan of Action for Non-Communicable Diseases (NCDs) Prevention and Control 2013–2020' aims to reduce mortality from these causes by 25% by 2025. Two of their nine global targets specifically prioritise preventing and controlling CVD:

- At least 50% of eligible people receive drug therapy and counselling to prevent heart attacks and strokes.
- A 25% relative reduction in the prevalence of raised blood pressure or contain the prevalence of raised blood pressure, according to national circumstances.

Given the complex and multifaceted nature of medication adherence, understanding patients' and caregivers' perspectives is crucial for designing effective interventions [13].While quantitative research provides valuable statistical insights into adherence rates and clinical outcomes, qualitative studies offer a deeper exploration of the lived experiences, beliefs, and contextual factors that influence medication adherence [19].

### Aim

The aim of this systematic review was to synthesise the available qualitative literature on the facilitators and barriers experienced by MI survivors and their families or caregivers in managing medications and adhering to treatment in order to inform the design of future behavioural interventions.

## Method

The reporting of this study followed the Preferred Reporting Items for Systematic Reviews and Meta-Analysis (PRISMA) checklist [20]. The review protocol was registered with the International Prospective Register of Systematic Reviews (PROSPERO) under the registration number CRD42023424844.

### Search strategy

The electronic databases PubMed, EMBASE, Web of Science, SCOPUS, and CINAHL were searched for relevant studies from their establishment until the 13th of August 2024. The search strategy was modified to align with the search capabilities of each database, in line with the PRISMA-S Checklist (see supplementary material) [20]. There were no constraints or limitations on the publication date or language. Reference lists of the included full texts were searched. Additionally, a citation search of the included full texts was conducted.

### Eligibility criteria

The PEO (People/Patient, Exposure, Outcome) statement was used to define the research criteria for the review [21]. Qualitative research articles were included to evaluate the views of people who have experienced MI regarding medication-taking behaviour post-discharge. The inclusion criteria were:

i. Post-MI patients aged ≥ 18 years old
ii. Discharge from the Emergency Department OR acute setting to home OR an independent care facility (a residential setting designed for individuals who need some level of assistance with daily activities but do not require full-time medical care).
iii. OR Family member OR caregiver for patients who meet the above criteria (i) and (ii).

Interviews, focus groups and mixed-methods studies with qualitative elements were considered. Quantitative research studies, studies that included patients younger than 18 or those discharged to another healthcare facility or care setting were deemed ineligible.

### Study selection

References retrieved from database searching were imported into Rayyan Software [22], and duplicates were removed. One author (FEK) screened all titles and abstracts for relevance, with a second independent screen performed by another author (MB, LS, or MOD). The remaining full texts were reviewed by two independent reviewers (FEK and either LS, MB, or MOD), with reasons for exclusion documented. At each stage, conflicts were resolved through discussion or the inclusion of a third researcher for arbitration if required.

### Quality assessment

The methodological quality of eligible studies was critically appraised, utilising the standard JBI Critical Appraisal Checklist for Qualitative Research tool for systematic reviews [23]. FEK conducted the critical appraisal, and a random 10% was independently checked by LS. Where conflicts arose, consensus was reached through discussion or consultation with an independent third reviewer.

### Data extraction

All data extraction and assessment were conducted by FEK and independently checked by a second reviewer (either LS, MB, or MOD), with consensus reached on any discrepancies. A data extraction template was used to collect the following information from each included paper: Study design, aim, participants, sample size, data collection method, analytical approach, and main findings.

### Data synthesis

The full-text articles were imported into NVivo-12 software for coding, using the Theoretical Domains Framework (TDF) to guide data extraction and synthesis [24]. The TDF is a comprehensive, validated framework comprising fourteen domains that encompass 84 theoretical constructs considered most pertinent to implementation inquiries. The framework consolidates multiple behaviour change theories, allowing for a structured approach to identifying key determinants of behaviour [18, 24–26]. This approach was chosen because it provides a robust method for analysing qualitative data in the context of behavioural interventions, thus ensuring that findings are mapped to established theoretical constructs. Additionally, the TDF can be linked to the Capability, Opportunity, Motivation-Behaviour (COM-B) model, allowing for the development of targeted interventions by identifying key capability, opportunity, and motivation factors influencing behaviour [27]. While the TDF can be complex in nature, data from the included studies were mapped only to relevant domains, ensuring that domains that emerged most strongly from the fourteen domains are presented in these findings.

The results/findings of each included article, including the entire text, themes, quotations, and tables, were independently coded by both FEK and MOD and consensus was reached on all discrepancies through discussion and mutual agreement. The identified codes were categorised into the relevant domains of the TDF and further assigned as either a barrier, a facilitator, or both barrier and facilitator to medication management and adherence post-MI.

The Confidence in the Evidence from the Reviews of Qualitative Research (CERQual) approach was applied to assess the confidence that may be placed on the findings from this qualitative evidence synthesis [28]. CERQual was used to enhance the transparency and reliability of the review findings. This approach assigns a confidence grade of high, moderate, low, or very low to the generated findings. The confidence grade for each discovery is determined by four components: the methodological limitations, relevance, coherence, and data adequacy of the included study. Confidence in review findings pertains to the probability that the review finding accurately reflects the studied topic. The confidence level starts at high and is downgraded according to judgements based on the four components. All tools were applied to all included studies by FEK, and the findings were checked by MOD.

## Results

### Search findings

**Fig. 1:**
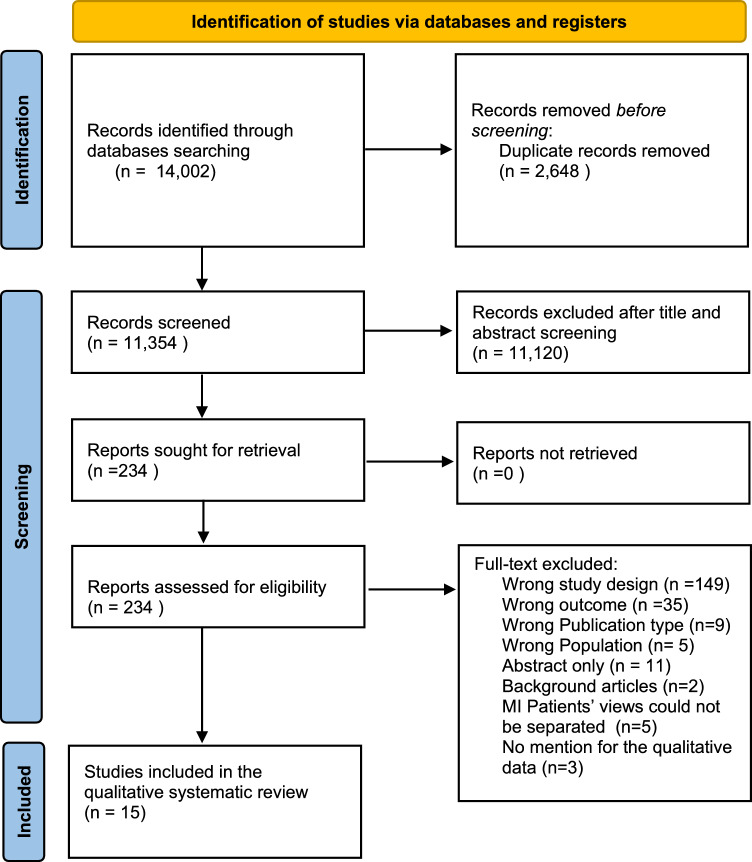
PRISMA flow chart diagram of the systematic review

The preliminary search yielded 14,002 titles. After resolving duplicates, 11,354 titles remained for title and abstract screening, resulting in 234 articles selected for full-text review (Fig. 1). Data were subsequently extracted from fifteen included articles.

### Characteristics of included studies

Table 1 shows the characteristics of the included studies. The studies included 533 people who had experienced MI and 25 spouses of such people. Studies were performed in eight different countries, including four studies from the United Kingdom (UK) [29–32]; three from Sweden [33–35]; two from Canada [36, 37]; two from the United States of America (USA) [38, 39]; one from Indonesia [40]; Iran [41]; Norway [42] and Northern Israel [43]. Fourteen studies were solely qualitative in design, and one study employed a mixed-methodology approach, incorporating qualitative feedback from participants through a free-text questionnaire section [32]. One study used a focus group methodology, with interviews used in 13 studies and text was used in one-study. [29–31, 33–43].

**Table 1:** Characteristics of included studies

Author, year, and location	Study Design	Participant(s)	Study aim	Method(s)	Analysis	Finding(s)	Key recommendations
White et al. United Kingdom [29]	Qualitative study	UK District General Hospital-Based Cardiac Rehabilitation (CR) Program 11 males, 4 females	Examines the practical use of the ‘medicines resistance’ model based on CR patients’ experiences with medicine-taking	Initial interviews were conducted three months after the completion of the CR programme	Grounded theory	Patients passively accepted medicines at first, but some did not follow up, making acceptance unclear. Others actively questioned conflicting information, CHD-related uncertainties, and dosage concerns	The 'medicines resistance' paradigm may pose challenges for patients, but focusing on medication adherence can help patients resolve their unease without solely interpreting it
Hanna et al. Northern Israel [43]	A phenomenological approach	Two different CR Northern Israel, 20 males, 2 females	To gain insights into the perceptions that underline health-related adherence behaviours from the perspective of patients who experienced a heart attack	Semi-structured Interview Guide for Cardiac Rehabilitation	Used content analysis method: TDF	Intrinsic factors like well-being, self-competency, and determination influence adherence, while habit changes remain challenging Extrinsic factors, including MI risk, family, and healthcare support, help reinforce lifestyle changes	The motivational theory aids adherence: cardiac care should include tailored strategies, shared care plans, and follow-ups. Future research should assess their effectiveness
Khoiriyati et al. Indonesia [40]	A phenomenological approach	Two private hospitals in Yogyakarta, Indonesia, 12 males and 3 females	Explore the barriers to patients’ transition experiences from hospital to homecare in post-acute coronary syndromes	Semi-structured Interviews	Thematic content analysis	Barriers to medication adherence include limited knowledge of alternatives, side effects, and psychological obstacles. Adherence is higher after a first heart attack but lower in recurrent cases	Acute Coronary Syndrome (ACS) Patients' Discharge Challenges:Need for enhanced communication, education, and preparedness.Proposed further research to mitigate these obstacles
Decker et al. USA [38]	Qualitative Study	TRIUMPH study: 11 stopped clopidogrel, 11 continued, matched by sex and education	Compares perceived barriers to medication adherence between patients who stop and those who continue life-sustaining medication	Telephone Interview Process,(Patient calls average 1.5 attempts)	Identified patterns and themes using the Human Behaviour Model	Patient perception of MI severity affects medication adherence Discontinuers delay treatment and feel vulnerable, while continuers focus on symptoms and recall medication details	Clopidogrel discontinuation was linked to system gaps; education is key. Continuers received better medication guidance
Dreyer et al. USA [39]	Exploratory qualitative study using a participatory action research (PAR) phenomenological approach	The study, conducted at multiple cardiovascular health centres, focuses on 42 patients with a history of first-time acute myocardial infarction	Gain a deeper insight into personal recovery in patients with AMI	Semi-structured interview up to 24 months post-discharge	Generated themes using the consensual model	Participants with AMI felt like a "different person," facing both losses and gains that challenged daily life. They took responsibility for their health, actively engaging in recovery and lifestyle changes	The study highlights the identity shift individuals experience due to AMI, highlighting the potential of the personal recovery framework in cardiology to underpin innovation in knowledge, roles, and interventions
Valaker et al. Norway [42]	Inductive exploratory design	Tertiary Percutaneous Coronary Intervention (PCI) centre in Western Norway, 22 discharged PCI Patients	To explore how patients undergoing PCI experience continuity of care between secondary and primary care settings after early discharge	Semi-structured Interview Guide Development	Qualitative content analysis, a hermeneutic interpretive paradigm	Patient's Challenges Post-Treatment, Lack of information on daily living and inability to make decisions due to illness	The study highlighted challenges in high-tech cardiac care, including issues with information, management, and continuity. It emphasized the need for improved care planning, teamwork, and nurse-led initiatives to enhance patient experience and care continuity
Desveaux et al. Canada [36]	A qualitative evaluation embedded within a multicentre randomised trial	Cardiac Study in Ontario, Canada, Nine cardiac centres, 17 males, 13 females	The study examines how well the intervention supports adherence to CR and cardiovascular medications, explores its mechanisms of action, and identifies ways to improve future interventions for patient needs	Phone Interviews (Questions informed by Health Action Process Approach and TDF)	Theory of Determinants of Disease domains and inductive coding	The study found that beliefs about capabilities and consequences influenced adherence. While motivational interviews helped establish intent, many patients did not complete CR or adhered inconsistently. Non-adherence was linked to emotions, identity, and external factors like inactivity, as well as challenges with medication and recall	Adherence after a cardiac event is influenced by beliefs about consequences, self-efficacy, intentions, social influences, and emotions. A multicomponent intervention using education and reminders supports CR adherence through reinforcement, behavioural regulation, knowledge, and social influence
Presseau et al. Canada [37]	Study 1: Qualitative interviews using TDF	20 males and 4 females	Compare the utility of different behaviour theory-based approaches for identifying modifiable determinants of medication adherence post-MI that interventions could target	Patients interviewed at various post-MI intervals (0–2, 3–12, 13–24, or 25–36 weeks post-MI)	Treatment Adherence Beliefs StudyParticipants' responses were coded into 14 TDF domains.	Beliefs about side effects and discontinuation, forgetfulness or intentional stopping of medications, and the establishment of routines and reminders influenced medication adherence. Trust in healthcare providers and family support were key factors, while friends' influence was less clear	Two theoretical approaches show that action planning and social support are key predictors of medication adherence post-MI, while self-efficacy and outcome expectations are key in intention. Novel theory-based interventions could help maximize recovery
Attebring et al. Sweden [33]	Qualitative study	University Hospital, Western Sweden, outpatient Clinic Study, 12 males, 8 females	To explore patients' experiences of secondary prevention after a first AMI	Interviews were conducted in patients' homes and hospital rooms, lasting 30–80 min, 7.5 weeks post-discharge	Hermeneutic Approach in Cardiac Care Analysis	Heart attack patients often mistake symptoms for medication side effects and worry about costs, dosages, and discomfort, sometimes enduring pain without treatment. Conflicting medical advice causes confusion, leading them to seek information online and reassurance from multiple sources. Aftercare challenges contribute to anxiety and insecurity in recognizing heart condition symptoms	Health professionals should consider the impact of treatment on patients, especially after a first MI. Secondary prevention should focus on patients' beliefs, and physicians should discuss long-term consequences from the patient’s perspective
Karner et al. Sweden [34]	Qualitative with an empirical and inductive approach	25 selected spouses, 17 females and 8 males	To explore spouses' conceptions concerning causes of coronary heart disease and drug treatment 1 year after the partner's cardiac event	Interviews with CR nurse and author, and surveyed views on prescribed drugs	Utilized phenomenon-graphic procedure	Three categories: (A) Drugs prevent atherosclerosis, clot formation, MI, and the need for a bypass operation. Spouses linked specific drugs like aspirin to pathophysiological aspects, believing it facilitates blood circulation, eases heart work, and prevents clot formation. (B) Drugs are necessary for the heart but harmful to other organs. Drugs are necessary for heart health, but they also carry risks of dependency and drug resistance; spouses find treatment helpful but also see it as inconvenient. (C) Constant drug intake damages function and leads to disease. The spouses expressed fear of long-term drug damage, focusing on damage to various functions or disease development as the primary cause of treatment. B was the most common answer	Spouses' misconceptions about CHD causes and drug treatment may impact patient cooperation with treatment and lifestyle changes, as spouse support is crucial for patients' cooperative attitude towards CHD-preventing strategies
Liljeroos et al. Sweden [35]	Qualitative study using thematic analysis	Uppsala University Hospital, Sweden, 9 Patients	To explore the self-perceived cognitive status and cognitive challenges associated with lifestyle changes in cardiac rehabilitation among elderly myocardial infarction (MI) patients (≥ 65 years)	Conducted semi-structured interviews 6–12 weeks post-MI via phone or in person	Authors coded and developed categories separately to minimise bias	Cognitive decline after MI affects memory, concentration, and planning, impacting self-care and rehabilitation. Barriers include low motivation, physical challenges, and medication side effects. Poor healthcare communication and limited access further hinder recovery, with patients advocating for better physician access, digital tools, and improved support materials	The study highlights how elderly MI patients normalise cognitive decline and often overlook their MI experiences. It emphasises the need for better patient-caregiver communication, treatment adherence, and coping strategies in rehabilitation. Optimising post-MI care at local health centers is crucial, with future research focusing on larger samples and caregiver perspectives using mixed methods
Jalal et al. United Kingdom [44]	A mixed method study is part of an original pilot randomised study	A London Heart Attack Centre 71 patients (54 non-Asians and 17 SAs). 13 males and 1 female	Investigate beliefs and experiences of South Asian patients regarding coronary heart disease and medication-taking behaviour	Interviews on Medicine-Taking Behaviour in Coronary Heart Disease Patients	Developed coding framework for interview schedules	Themes were derived from the results: Disease Perception: Patients reported pain and believed the disease was cured after a procedure; some cited genetics or diet as causes Medicine Perception: Medicines are important, but concerns about safety exist; half rely on others to remember medications Adherence Factors: Forgetfulness, family support, and side effects like muscle pain impact adherence	This article examines secondary prevention after myocardial infarction in South Asian (SA) patients, focusing on behaviour, adherence to medication, living with the disease, and cardiac rehabilitation. It found similar adherence patterns in SA and non-Asian patients, but older SAs perceived the disease as acute, while younger patients struggled
Webster et al. United Kingdom [31]	Qualitative research approach	Survey on Gujarati Hindu MI Patients, admitted Leicester's coronary care units 25 males and 10 females born in India but spent at least 3 years living in the UK	To explore the experiences and needs of Gujarati Hindu patients and their partners in the first month after a myocardial infarction	Utilized semi-structured interviews two to three weeks post-discharge	Coded and compared interviews for broader categories, constant comparative method was used for data comparison	Patients and their partners lacked clarity about their diagnosis and recovery, with few recalling receiving information or visits from a cardiac rehabilitation nurse. Written materials in English were often unread, leading to confusion about rehabilitation. Many mistook it for an exercise test or outpatient appointment. Home visits from the research assistant were appreciated, as were Gujarati-speaking nurses in hospitals	The study of a homogeneous south Asian group revealed a lack of knowledge and beliefs about MI, leading to poor adherence to cardiac rehabilitation programs and secondary prevention strategies. The study suggests that effective treatment depends on cultural awareness, language issues, and social and family circumstances. However, social class and standard of living may also play a role
Khatib et al. United Kingdom [32]	Single-centre, non-comparative mixed methods study design	138 males and 66 females, with a total of 86 patients, used the free text box to add additional comments	This study describes the rationale and development of the My Experience of Taking Medicines (MYMEDS) tool	MYMEDS Adherence Scale Design Principles: The last section allows the Patient to write free text Feedback on Medicine-Taking Experience	Only answers related to MYMEDS were included in the analysis	The MYMEDS questionnaire's free-text responses highlight key concerns. Patients worry about specific and potential side effects, as well as issues related to tablet size, swallowing, and administration instructions. Concerns about drug and food interactions are common, along with questions about the necessity of medications when health parameters appear normal. Patients also report challenges with prescriptions and seek more information on side effects and treatment duration. Additionally, caregivers play a crucial role in managing medications	MYMEDS is a practical tool that effectively identifies and modifies barriers to SPM adherence, making it easy to implement in clinical practice for better patient focus
Nadery et al. Iran [41]	Conventional qualitative content analysis	16 males and females including 12 nurses, 2 doctors, and 2 patients	To identify the causes of nonadherence in people with myocardial infarction	13 Interviews and a Focus Group was Conducted	Content analysis performed on each interview; data analysed using Max QD software	Drug-related issues like insurance and distribution can impact access and adherence. Cultural factors, including stigma, reluctance, and reliance on nonexpert advice, also affect treatment. Patients struggle with adherence due to mental health issues, scepticism about medical advice, and misconceptions about recovery. Many discontinue medication when feeling relatively well, emphasizing the need for better understanding and support	Nonadherence to treatment regimens can be attributed to individual characteristics and healthcare organizations, requiring awareness and appropriate solutions

These studies assessed patients'/carers' perspectives on managing and adhering to medications post-MI. All fourteen domains of the TDF were reported as either a barrier, a facilitator, or both, as they pertained to medication adherence. The five most prominent domains are presented in Table 2 in addition to illustrative quotes.

**Table 2:** Summary of findings regarding barriers and facilitators for medication adherence and management post-MI

TDF domain	TDF sub-domain	Patient quotes
Beliefs about consequences	Consequences of non-adherence	“Yes, I consider it serious, yes… I think it is going to limit my life from here.”[38]
	Family beliefs	“I suppose that the drugs mean that this situation does not arise again…and then you’ll be spared a new operation and heart attack…it’s for the sake of the disease, to keep it down, and that’s no disadvantage”[34]
	Adherence consequences	“I am concerned about side effects of the medications”[37] “……One is sent home with a lot of drugs. Hitherto, the only medication I took was the odd antibiotics, but all of a sudden, I have to balance six or seven drugs with all their implications of side effects and things like that.”[33]
Environmental context and resource:	Resources	“Some drugs does not include insurance or pharmacies do not have a contract with this insurance.”[41] “I’m always getting confused when I meet the doctor. Then I forget what to ask about. I think this has to do with the restricted time you have. You have only 10 min. First, you sit and wait for half an hour or three-quarters. So when you enter the doctor’s room, you get a feeling of getting out quickly and the doctors have too little time with you.”[42]
Social Influence	Family support	“I mean my family took care of me. I was emotional at times because I’m so used to doing things myself. So that got to me a little bit. My husband and my son, they did everything, took care of me, watched over me, sometimes too much.”[39] “I don’t like people telling me what to do. I know what is best for me and what not. I am not a young child. I know exactly what I should do.”[43]
	Healthcare professionals	“I take medication because my healthcare providers recommend it.”[37] “The doctor said they are important for my body, I do not think about it if doctor says I need it then I need it that’s it”[44]
Emotion	Fear and stigma	“If I take medicine, I feel sick, but when I’m not taking medicine and going to the club, I’m feeling better mentally and I think I’m healthier.”[41] Another patient felt that the medication was limiting his daily activities “I feel like the medication is inhibiting the… During the first part of the day, so to say, when you’re active”[35]
	Optimism	“So, I think in a sense to me, that was my wake-up call, …I could have died then, but I didn’t, so there’s a reason. I don’t know. I’m just lucky to be alive. That day, I quit smoking, never another cigarette. Switched my diet…. I kept thinking, everything happens for a reason, and things could have been a lot worse.”[33]
Knowledge	Lack of knowledge	“I didn’t hear anything from the rehabilitation. And then I thought, it has been Pentecost and the 17th of May, but then I called them. And it was a good thing that I did because they had forgotten to refer me.”[42] “It worried me taking eight different types of tablets” because “it does say in the book that the most tablets patients would normally be on is three or four tablets a day.”[29]
	Having knowledge	One spouse pointed out the importance of aspirin use; “It sort of has the effect of dissolving clots. It thins the blood a bit, not like Waran ~ (anticoagulant) it’s not, but…Trombyl ~ (aspirin) it’s so it won’t form clots”[34] Another patient reported that her son received the necessary information for her medication before discharge; “Yes, my GP told my son, and at the hospital they told my son this is for this and this is for that, but they are important to take”[44]

### Domain 1—Beliefs about consequences

This was the most mentioned domain across the 15 studies. Most patients considered medicines to be crucial for their health, with some expressing concerns about safety due to the high number of tablets required. Patients took their medications because they believed that MI was a serious condition that might lead to more complications. Family members reflected on their beliefs regarding the importance of medication adherence in preventing further complications and acknowledged the significant role that adherence plays in achieving positive health outcomes. Patients often struggled with adherence to medication after experiencing severe heart pain. This could have been due to various factors, including a lack of belief in medical advice and a lack of confidence in the drugs. Patients were sometimes also in denial about the disease, and incorrect beliefs could negatively affect medication adherence, with some patients expressing concern regarding medication side effects.

### Domain 2—Environmental context and resource

Patients faced numerous contextual challenges relating to medication taking. This included insufficient communication channels with their treatment teams post-discharge, which hindered proper recovery and management. Patients often encountered improper behaviour and inadequate hospital facilities, impacting their overall care experience. Poor insurance coverage for necessary paraclinical actions and medications also exacerbated the financial burden, especially for disadvantaged populations.

Adherence to treatment regimens was also challenged by disruptions to daily lives, job-related barriers, and a lack of timely and adequate education on managing their condition. An often-cited obstacle was communication failure, resulting in inadequate treatment transitions, such as from inpatient to outpatient or specialist to primary care physician. Conversely, one patient characterised the hospital discharge process as being a cue for taking action, receiving adequate information regarding the medication and emphasising the importance of adherence.

### Domain 3—Social influence

#### Subdomain—Family support

Many participants living with their families found it easier to adhere to their medications. Family members, particularly spouses, offered valuable reminders to adhere to treatment, as well as practical assistance. Perceiving real concern and support from others was considered crucial for adherence. Conversely, some participants felt that family support added pressure, and they felt uncomfortable or even offended by others’ attitudes regarding their adherence.

#### Subdomain—Healthcare professionals

Healthcare professionals were reported to significantly influence medication adherence, as their advice and guidance could greatly impact a patient's willingness to follow prescribed treatment plans. Some patients seemed to have a strong tendency to trust their doctors' prescribing decisions and directions. Having conversations with healthcare professionals could positively impact medication management and adherence, for example, if the patient experienced side effects. On the contrary, some patients felt that the information received from healthcare professionals was conflicting, which led to uncertainty and confusion among patients.

### Domain 4—Emotion

Emotions reflected in the findings of the included studies encompassed the fear of being stigmatised due to heart disease and unwillingness to adhere to treatment. Individuals often preferred not to be perceived as cardiac patients. As a result, they sometimes concealed their condition and avoided taking medication in public settings. Sometimes, as a consequence of their participation in the study interview, patients realised the importance of their medicines, and this was reflected in planned behaviour. In contrast, others felt that the medication was limiting their daily activities. In addition, some showed concerns regarding the number of tablets taken by them or their partners. On the other hand, others felt that they had survived and that this was a second chance at life, so they chose to take advantage of it by leading a healthier life and adhering to their medication.

### Domain 5—Knowledge

Patients frequently mentioned their lack of knowledge regarding medications. This served as a barrier for many patients, as they did not understand the rationale for taking all of these medications and the potential side effects. Other barriers included (i) being unaware of the cardiac rehabilitation programs, (ii) a lack of written information, and (iii) the use of medical jargon when being provided with information.

Interestingly, several individuals were able to state the name and the use of each medication, while others showed little knowledge. However, most patients were interested in receiving information from healthcare professionals and emphasised the importance of having written support documentation. They acknowledged that having appropriate knowledge could positively influence someone’s decision to take their medication.

### Quality appraisal and CERQual grading

The fifteen included studies generally satisfied the JBI criteria for the quality of the included qualitative studies, for example, the congruity between the stated philosophical perspective and the chosen research methodology, as well as the consistency between the research methodology and the research question or objective [29, 31–44]. However, the criteria regarding locating the researcher culturally or theoretically were met in only four articles [35, 37, 41, 42]. Additionally, the researcher's influence on the research and vice-versa was reported in only two studies [38, 42]. Using the JBI checklist pertaining to methodology, a full description of the reporting assessment can be found in Table 3. The CERQual approach was used to assess confidence in our review findings based on methodological limitations, coherence, adequacy, and relevance [28]. All included studies showed high confidence, meeting the criteria across all components**.** In this review, the confidence level was not downgraded if any components were judged to have minor or very minor concerns**.**

**Table 3:** Quality appraisal according to JBI [23]

Author(s), Year	Attebring et al. [33]	Decker et al. [38]	Desveaux et al. [36]	Dreyer et al. [39]	Hanna et al. [43]	Jalal et al. [44]	Karner et al. [34]	Khatib et al. [32]	Khoiriyati et al. [40]	Liljeroos et al. [35]	Nadery et al. [41]	Presseau et al. [37]	Valaker et al. [42]	Webster et al. [31]	White et al. [29]
Is there congruity between the stated philosophical perspective and the research methodology?	Y	Y	Y	Y	Y	Y	Y	Y	Y	Y	Y	Y	Y	Y	Y
Is there congruity between the research methodology and the research question or objectives?	Y	Y	Y	Y	Y	Y	Y	Y	Y	Y	Y	Y	Y	Y	Y
Is there congruity between the research methodology and the methods used to collect data?	Y	Y	Y	Y	Y	Y	Y	Y	Y	Y	Y	Y	Y	Y	Y
Is there congruity between the research methodology and the representation and analysis of data?	Y	Y	Y	Y	Y	Y	Y	Y	Y	Y	Y	Y	Y	Y	Y
Is there congruity between the research methodology and the interpretation of results?	Y	Y	Y	Y	Y	Y	Y	Y	Y	Y	Y	Y	Y	Y	Y
Is there a statement locating the researcher culturally or theoretically?	N	N	N	N	N	N	N	N	N	Y	Y	Y	Y	N	N
Is the influence of the researcher on the research, and vice-versa, addressed?	N	Y	N	N	N	N	N	N	N	N	N	N	N	N	N
Are participants, and their voices, adequately represented?	Y	Y	Y	N	Y	Y	Y	Y	Y	Y	Y	Y	Y	Y	Y
Is the research ethical according to current criteria or, for recent studies, and is there evidence of ethical approval by an appropriate body?	Y	U	Y	Y	Y	Y	Y	Y	Y	Y	Y	Y	Y	Y	Y
Do the conclusions drawn in the research report flow from the analysis, or interpretation, of the data?	Y	Y	Y	Y	Y	Y	Y	Y	Y	Y	Y	Y	Y	Y	Y

Y: Yes, N: No, U: Unclear, NA: Not applicable

## Discussion

### Statement of the key findings

This qualitative systematic review utilised the TDF to explore the barriers and facilitators influencing medication adherence post-MI. The use of the TDF allowed for a structured and theory-informed synthesis of qualitative data, helping to identify key behavioural determinants relevant to medication adherence. The five most prominently featured TDF domains were; Beliefs about consequences, Environmental context and resources, Social influences, Emotion, and Knowledge.

### Strengths and weaknesses

The use of TDF in this review revealed complexity across all 14 TDF domains, highlighting the challenge of consistently implementing guideline-based care. The studies included in this review provide valuable insights into medication adherence, but collectively, their power lies in how they can be synthesised to guide development of an intervention. By applying the TDF and mapping these findings to the COM-B model, we can move beyond isolated findings to a broader understanding of how best to support adherence.

This review has explored barriers and facilitators to medication management and, thus, medication adherence post-MI across a range of geographical and cultural contexts. Its transferability is enhanced by the utilisation of CERQual to confirm our confidence in the findings. The added value of using the CERQual grading approach lies in its ability to assess the confidence of synthesised qualitative findings rather than the quality of individual studies. However, as highlighted by Wainwright M. et al., there is no consensus on using CERQual for individual study quality assessment, and this remains an area requiring further discussion and methodological clarity [45]. This review also included studies that used surveys with open comment sections as their qualitative method, ensuring maximal coverage of the qualitative data.

One key limitation is the broader methodological challenge inherent in systematic qualitative synthesis. There is currently no universally accepted approach for conducting a "systematic qualitative review" or qualitative meta-synthesis [46, 47]. Whilst the current study used the TDF to structure the analysis, alternative frameworks or synthesis methods could potentially yield different interpretations. Although this review involved a comprehensive search strategy, a potential limitation is the exclusion of studies whereby the views of patients could not be separated from those of healthcare professionals.

### Interpretation

This review identified several facilitators and barriers to medication adherence post-MI from both patient and carer perspectives. Among the facilitators, patients’ beliefs about the consequences of medication adherence emerged as the most prominent, followed by the significant influence of family members and social networks on adherence behaviours. Conversely, lack of knowledge about medications and concerns about their side effects were prominent barriers identified in the studies. Emotions related to fear or anxiety and conflicting information received from healthcare professionals also contributed significantly to reported non-adherence.

Importantly, while certain TDF domains served as facilitators for adherence in some patients, they acted as barriers for others, highlighting the need for personalised approaches in post-discharge care to enhance adherence rates. This finding is supported by a previous review of the adoption of prescribing guidelines and a further review of the implementation of physical activity policies in schools [48, 49].

It was found that different combinations of domains might be linked to or influence one another. For example, knowledge, or lack of knowledge, can both affect patients' beliefs about the consequences of medication adherence. The relationship between knowledge and beliefs about medication adherence is a crucial factor influencing patient behaviour, particularly in post-myocardial infarction care. A 2013 study highlighted that cardiac rehabilitation patients with limited knowledge of their medications often expressed resistance to adherence due to mistrust or a perceived lack of necessity [29]. This suggests that insufficient understanding of medications can lead to negative beliefs about their importance and efficacy [50]. In contrast, patients with better knowledge were more likely to view adherence positively, as they understood the health benefits and consequences of adhering to their medication regimen. This finding is echoed in another study, in 2020 where MI patients reported that adherence was significantly influenced by their understanding of the medication’s role in preventing future health complications. Patients who felt informed about their condition and treatment and who received support from healthcare providers were more likely to adhere to their prescribed medications. The same study described the importance of combining education with support to shape positive beliefs about the necessity and benefits of adherence [43]. Another study conducted in 2021 in Ethiopia demonstrates similar findings: patients who believe that their medication is important are more likely to adhere to their medication than those who believe it is harmful to their health. The evidence suggests that interventions aimed at enhancing knowledge and addressing misconceptions may be key to overcoming barriers to adherence [51]. Comprehensive education strategies, coupled with supportive healthcare environments, are vital in ensuring patients have the necessary understanding to form beliefs that promote adherence [52].

One of the reported interventions provided patients with written information regarding their medications. This aided their understanding of the medications and, thus, adherence [32, 42]. A qualitative study published in 2024 in three urban districts in China found that providing various forms of information and using digital health tools enhanced medication management and adherence [53]. Another method of delivering information mentioned by the patients in several studies was engaging in a conversation with a healthcare professional to understand the potential side effects of their medication and the reason for taking these prescribed medications [29, 38, 44]. A Finnish study published in 2018 on the role of healthcare providers in medication adherence found that better communication, particularly discussing the necessity and safety of medications, was linked to higher adherence rates [54].

Family support can result in negative and/or positive impacts. These findings align with existing research that shows how social support, particularly from family, generally enhances patients' adherence. However, a study conducted in 2004 also found that excessive involvement or criticism from family members could lead to non-adherence [55]. Similarly, a study conducted in Spain and published in 2019 found that while family involvement typically improves adherence, over-involvement or undue pressure can negatively impact a patient’s willingness to follow their treatment plan [56]. Another review and meta-synthesis published in March 2022 highlighted the crucial role of family members in supporting lifestyle changes for individuals with prediabetes, emphasising their role in providing motivation and practical assistance. Their findings suggest that family support can help individuals overcome barriers to adopting and maintaining healthier behaviours [57].

The TDF enabled the synthesis of findings across multiple studies in a way that identifies the most common themes that influence medication adherence behaviour. Importantly, future mapping of these findings to the capability, opportunity, and motivation model (COM-B), the three core components driving behaviour change, will allow this evidence to be translated into actionable intervention strategies [58]. This integrative approach enhances our understanding of adherence challenges and informs the development of targeted, theory-driven, and evidence-based interventions. By highlighting key considerations for individualised patient care, the findings emphasise the importance of personalised strategies to improve medication adherence post-MI, ultimately leading to better patient outcomes.

### Future research

Future research should continue to refine and compare qualitative synthesis methodologies to establish best practices in this evolving field. Our approach offers a useful model for understanding the behavioural determinants of adherence and can inform the design of future interventions. Further studies could explore how to harness the benefits of family support while minimising its potential drawbacks. This might involve developing programs that train family members in effective communication and support techniques or tools that help patients and their families set healthy boundaries, promoting adherence without increasing stress. Such work would enhance our understanding of the family’s role in health management and support the development of more personalised and effective care strategies.

## Conclusion

Whilst optimal medication management and adherence are crucial for better outcomes post-MI, this review highlights the most prominent TDF domains that affect adherence. These are “Beliefs about consequences”, “Environmental context and resources”, “Social influence” and finally “Emotion and knowledge”. This review highlights the need for interventions that address the specific behavioural drivers identified, ensuring a more comprehensive and effective approach to improving medication adherence. Tailoring interventions to address specific barriers and leveraging facilitators identified in this review could significantly improve medication adherence post-MI and subsequently enhance patient outcomes.

## Supplementary Information

Below is the link to the electronic supplementary material.

Supplementary file1 (DOCX 42 kb)

## References

[1] Cardiovascular Diseases (Cvds). World Health Organization, World Health Organization. 2021. www.who.int/news-room/fact-sheets/detail/cardiovascular-diseases-(cvds). Accessed 1 Feb 2025

[2] Thygesen K, Alpert JS, Jaffe AS, et al. Fourth universal definition of myocardial infarction. Eur Heart J. 2018;40:237–69. 10.1093/eurheartj/ehy462. 10.1093/eurheartj/ehy462 30165617

[3] Byrne RA, Rossello X, Coughlan JJ, et al. ESC guidelines for the management of acute coronary syndromes: developed by the task force on the management of acute coronary syndromes of the European society of cardiology (ESC). Eur Heart J. 2023;44:3720–826. 10.1093/eurheartj/ehad191. 37622654 10.1093/eurheartj/ehad191

[4] Kolansky DM. Acute coronary syndromes: morbidity, mortality, and pharmacoeconomic burden. Am J Manag Care. 2009;15:S36-41. 19355807

[5] Salari N, Morddarvanjoghi F, Abdolmaleki A, et al. The global prevalence of myocardial infarction: a systematic review and meta-analysis. BMC Cardiovasc Disord. 2023;23:206. 37087452 10.1186/s12872-023-03231-w PMC10122825

[6] Gabulova R, Marzà-Florensa A, Rahimov U, et al. Risk factors in cardiovascular patients: challenges and opportunities to improve secondary prevention. World J Cardiol. 2023;15:342–53. 37576543 10.4330/wjc.v15.i7.342 PMC10415862

[7] Fuller RH, Perel P, Navarro-Ruan T, et al. Improving medication adherence in patients with cardiovascular disease: a systematic review. Heart Br Card Soc. 2018;104:1238–43. 10.1136/heartjnl-2017-312571 29572248

[8] Naderi SH, Bestwick JP, Wald DS. Adherence to drugs that prevent cardiovascular disease: meta-analysis on 376,162 patients. Am J Med. 2012;125:882-887.e1. 22748400 10.1016/j.amjmed.2011.12.013

[9] De Geest S, Sabaté E. Adherence to long-term therapies: evidence for action. Eur J Cardiovasc Nurs. 2003;2:323–323. 10.1016/S1474-5151(03)00091-4. 14667488 10.1016/S1474-5151(03)00091-4

[10] De Geest S, Ruppar T, Berben L, et al. Medication non-adherence as a critical factor in the management of presumed resistant hypertension: a narrative review. EuroIntervention. 2014;9:1102–9. 10.4244/EIJV9I9A185. 24457281 10.4244/EIJV9I9A185

[11] Shin J, McCombs JS, Sanchez RJ, et al. Primary nonadherence to medications in an integrated healthcare setting. Am J Manag Care. 2012;18:426–34. 22928758

[12] Olivieri NF, Matsui D, Hermann C, et al. Compliance assessed by the medication event monitoring system. Arch Dis Child. 1991;66:1399–402. 1776885 10.1136/adc.66.12.1399 PMC1793394

[13] Ho PM, Bryson CL, Rumsfeld JS. Medication adherence: its importance in cardiovascular outcomes. Circulation. 2009;119:3028–35. 19528344 10.1161/CIRCULATIONAHA.108.768986

[14] Simpson SH, Eurich DT, Majumdar SR, et al. A meta-analysis of the association between adherence to drug therapy and mortality. BMJ. 2006;333:15. 16790458 10.1136/bmj.38875.675486.55 PMC1488752

[15] Horwitz RI, Horwitz SM. Adherence to treatment and health outcomes. Arch Intern Med. 1993;153:1863–8. 8250647

[16] Osterberg L, Blaschke T. Adherence to medication. N Engl J Med. 2005;353:487–97. 16079372 10.1056/NEJMra050100

[17] Rosenbaum L. Beyond belief–how people feel about taking medications for heart disease. N Engl J Med. 2015;372:183–7. 25564902 10.1056/NEJMms1409015

[18] Roth GA, Johnson C, Abajobir A, et al. Global, regional, and national burden of cardiovascular diseases for 10 causes, 1990 to 2015. J Am Coll Cardiol. 2017;70:1–25. 28527533 10.1016/j.jacc.2017.04.052 PMC5491406

[19] Tong A, Sainsbury P, Craig J. Consolidated criteria for reporting qualitative research (COREQ): a 32-item checklist for interviews and focus groups. Int J Qual Health Care J Int Soc Qual Health Care. 2007;19:349–57. 10.1093/intqhc/mzm042 17872937

[20] Page MJ, McKenzie JE, Bossuyt PM, et al. The PRISMA 2020 statement: an updated guideline for reporting systematic reviews. BMJ. 2021. 10.1136/bmj.n71. 33782057 10.1136/bmj.n71 PMC8005924

[21] Teesside University, Student and Library Services. Developing Your Search Question using PICO/PIO/PEO. 2025. Available from: https://www.tees.ac.uk/lis/learninghub/cinahl/pico.pdf Accessed 1 Feb 2025

[22] Ouzzani M, Hammady H, Fedorowicz Z, et al. Rayyan—a web and mobile app for systematic reviews. Syst Rev. 2016;5:210. 10.1186/s13643-016-0384-4. 27919275 10.1186/s13643-016-0384-4 PMC5139140

[23] Munn Z, Barker TH, Moola S, et al. Methodological quality of case series studies: an introduction to the JBI critical appraisal tool. JBI Evid Synth. 2020;18:2127. 33038125 10.11124/JBISRIR-D-19-00099

[24] Michie S, Johnston M, Abraham C, et al. Making psychological theory useful for implementing evidence based practice: a consensus approach. Qual Saf Health Care. 2005;14:26–33. 15692000 10.1136/qshc.2004.011155 PMC1743963

[25] Cane J, O’Connor D, Michie S. Validation of the theoretical domains framework for use in behaviour change and implementation research. Implement Sci. 2012;7:37. 10.1186/1748-5908-7-37. 22530986 10.1186/1748-5908-7-37 PMC3483008

[26] Atkins L, Francis J, Islam R, et al. A guide to using the Theoretical Domains Framework of behaviour change to investigate implementation problems. Implement Sci. 2017;12:77. 10.1186/s13012-017-0605-9. 28637486 10.1186/s13012-017-0605-9 PMC5480145

[27] Michie S, van Stralen MM, West R. The behaviour change wheel: a new method for characterising and designing behaviour change interventions. Implement Sci. 2011;6:42. 10.1186/1748-5908-6-42 PMC3096582 21513547

[28] Lewin S, Glenton C, Munthe-Kaas H, et al. Using qualitative evidence in decision making for health and social interventions: an approach to assess confidence in findings from qualitative evidence syntheses (GRADE-CERQual). PLoS Med. 2015;12: e1001895. 26506244 10.1371/journal.pmed.1001895 PMC4624425

[29] White S, Bissell P, Anderson C. A qualitative study of cardiac rehabilitation patients’ perspectives on taking medicines: implications for the “medicines-resistance” model of medicine-taking. BMC Health Serv Res. 2013. 10.1186/1472-6963-13-302. 23938083 10.1186/1472-6963-13-302 PMC3750914

[30] Jalal Z, Paudyal V, Al-Arkee S, et al. Designing a clinical pharmacy primary care intervention for myocardial infarction patients using a patient and public involvement discussion. Pharm. 2020;8:13. 10.3390/pharmacy8010013 PMC7151658 31991672

[31] Webster RA, Thompson DR, Mayou RA. The experiences and needs of Gujarati Hindu patients and partners in the first month after a myocardial infarction. Eur J Cardiovasc Nurs. 2002;1:69–76. 14622870 10.1016/S1474-5151(01)00005-6

[32] Khatib R, Patel N, Hall AS. The my experience of taking medicines (MYMEDS) questionnaire for assessing medicines adherence barriers in post-myocardial infarction patients: development and utility. BMC Cardiovasc Disord. 2020. 10.1186/s12872-020-01362-y. 32013880 10.1186/s12872-020-01362-y PMC6998082

[33] Attebring MF, Herlitz J, Ekman I. Intrusion and confusion—the impact of medication and health professionals after acute myocardial infarction. Eur J Cardiovasc Nurs. 2005;4:153–9. 15904886 10.1016/j.ejcnurse.2005.02.001

[34] Karner A, Dahlgren M, Bergdahl B. Coronary heart disease: causes and drug treatment - spouses’ conceptions. J Clin Nurs. 2004;13:167–76. 14723668 10.1046/j.1365-2702.2003.00871.x

[35] Liljeroos T, Puthoopparambil SJ, Wallert J, et al. Self-perceived cognitive status and cognitive challenges associated with cardiac rehabilitation management: experiences of elderly myocardial infarction patients. Disabil Rehabil. 2022;44:3834–42. 33621136 10.1080/09638288.2021.1888321

[36] Desveaux L, Saragosa M, Russell K, et al. How and why a multifaceted intervention to improve adherence post-MI worked for some (and could work better for others): an outcome-driven qualitative process evaluation. BMJ Open. 2020;10:e036750. 32883724 10.1136/bmjopen-2019-036750 PMC7473621

[37] Presseau J, Schwalm JD, Grimshaw JM, et al. Identifying determinants of medication adherence following myocardial infarction using the theoretical domains framework and the health action process approach. Psychol Health. 2017;32:1176–94. 27997220 10.1080/08870446.2016.1260724

[38] Decker C, Garavalia L, Garavalia B, et al. Clopidogrel-taking behavior by drug-eluting stent patients: discontinuers versus continuers. Patient Prefer Adher. 2008;2:167–75. 10.2147/ppa.s3443 PMC2770390 19920959

[39] Dreyer RP, Pavlo AJ, Horne A, et al. Conceptual framework for personal recovery in patients with acute myocardial infarction. J Am Heart Assoc. 2021;10: e022354. 34581198 10.1161/JAHA.121.022354 PMC8649153

[40] Khoiriyati A, Sukartini T, Kusnanto K, et al. Barriers in the process of care transition from hospital to home in post-acute coronary syndrome: patients’ perspective. Bali Med J. 2021;10:1308–12.

[41] Nadery Y, Khorasani P, Feizi A, et al. Causes of nonadherence to treatment in people with myocardial infarction: content analysis. J Educ Health Promot. 2021;10:330. 34761016 10.4103/jehp.jehp_92_21 PMC8552253

[42] Valaker I, Norekvål TM, Råholm M-B, et al. Continuity of care after percutaneous coronary intervention: the patient’s perspective across secondary and primary care settings. Eur J Cardiovasc Nurs. 2017;16:444–52. 28111970 10.1177/1474515117690298 PMC5458873

[43] Hanna A, Yael E-M, Hadassa L, et al. ``It’s up to me with a little support’’ – adherence after myocardial infarction: a qualitative study. Int J Nurs Stud. 2020;101:103416. 31670171 10.1016/j.ijnurstu.2019.103416

[44] Jalal Z, Antoniou S, Taylor D, et al. South Asians living in the UK and adherence to coronary heart disease medication: a mixed- method study. Int J Clin Pharm. 2019;41:122–30. 30564971 10.1007/s11096-018-0760-3 PMC6394505

[45] Wainwright M, Zahroh RI, Tunçalp Ö, et al. The use of GRADE-CERQual in qualitative evidence synthesis: an evaluation of fidelity and reporting. Health Res Policy Syst. 2023;21:77. 10.1186/s12961-023-00999-3. 37491226 10.1186/s12961-023-00999-3 PMC10369711

[46] Flemming K, Noyes J. Qualitative evidence synthesis: where are we at? Int J Qual Methods. 2021;20:1609406921993276. 10.1177/1609406921993276.

[47] Soilemezi D, Linceviciute S. Synthesizing qualitative research: reflections and lessons learnt by two new reviewers. Int J Qual Methods. 2018;17:1609406918768014. 10.1177/1609406918768014.

[48] Paksaite P, Crosskey J, Sula E, et al. A systematic review using the theoretical domains framework to identify barriers and facilitators to the adoption of prescribing guidelines. Int J Pharm Pract. 2021;29:3–11. 32656929 10.1111/ijpp.12654

[49] Nathan N, Elton B, Babic M, et al. Barriers and facilitators to the implementation of physical activity policies in schools: a systematic review. Prev Med. 2018;107:45–53. 29155228 10.1016/j.ypmed.2017.11.012

[50] Neame R, Hammond A. Beliefs about medications: a questionnaire survey of people with rheumatoid arthritis. Rheumatol Oxf Engl. 2005;44:762–7. 10.1093/rheumatology/keh587 15741193

[51] Kasahun AE, Sendekie AK, Mekonnen GA, et al. Impact of personal, cultural and religious beliefs on medication adherence among patients with chronic diseases at university hospital in Northwest Ethiopia. Patient Prefer Adherence. 2022;16:1787–803. 35923657 10.2147/PPA.S370178 PMC9342701

[52] Bhattad PB, Pacifico L. Empowering Patients: Promoting Patient Education and Health Literacy. Cureus. 2022;14: e27336. 36043002 10.7759/cureus.27336 PMC9411825

[53] Feng Z, Li H, Chen X, et al. Patient participation in medication safety for noncommunicable diseases: a qualitative study of general practitioners, pharmacists, and outpatients’ perspectives in Beijing. Patient Prefer Adher. 2024;18:1907–18. 10.2147/PPA.S474921 PMC11409925 39296427

[54] Kvarnström K, Airaksinen M, Liira H. Barriers and facilitators to medication adherence: a qualitative study with general practitioners. BMJ Open. 2018;8:e015332. 29362241 10.1136/bmjopen-2016-015332 PMC5786122

[55] DiMatteo MR. Social support and patient adherence to medical treatment: a meta-analysis. Health Psychol Off J Div Health Psychol Am Psychol Assoc. 2004;23:207–18. 10.1037/0278-6133.23.2.207 15008666

[56] Fernandez-Lazaro CI, García-González JM, Adams DP, et al. Adherence to treatment and related factors among patients with chronic conditions in primary care: a cross-sectional study. BMC Fam Pract. 2019;20:132. 10.1186/s12875-019-1019-3. 31521114 10.1186/s12875-019-1019-3 PMC6744672

[57] Skoglund G, Nilsson BB, Olsen CF, et al. Facilitators and barriers for lifestyle change in people with prediabetes: a meta-synthesis of qualitative studies. BMC Public Health. 2022;22:553. 10.1186/s12889-022-12885-8. 35313859 10.1186/s12889-022-12885-8 PMC8935766

[58] Keyworth C, Epton T, Goldthorpe J, et al. Acceptability, reliability, and validity of a brief measure of capabilities, opportunities, and motivations (“COM-B”). Br J Health Psychol. 2020;25:474–501. 32314500 10.1111/bjhp.12417

