# Supplementary material for: Barriers and facilitators to managing medicines at home post-myocardial infarction: a qualitative systematic review
Source: Int J Clin Pharm. 2025 Jun 4;47(6):1549–66. doi: 10.1007/s11096-025-01927-x (PMC12630175; doi:10.1007/s11096-025-01927-x)
Supplement: Supplementary file 1 — Supplementary file1 (DOCX 42 kb) [file 11096_2025_1927_MOESM1_ESM.docx]

| **Supplementary Material 1: PRISMA-S for search strategy** | |
| --- | --- |
| **Checklist Item** | **Description** |
| **Information sources and methods** | |
| Database name | The following electronic databases were searched: PubMed, Embase, Web of Science, SCOPUS and CINAHL. |
| Multi-database searching | Not applicable; searches were run on databases individually. |
| Study registries | Not applicable. |
| Online resources and browsing | Not applicable. |
| Citation searching | Reference lists of all included full-text articles were manually screened to identify additional studies. |
| Contacts | The corresponding author of the following studies was contacted to obtain clarification on aspects of studies or to obtain full text papers:  **Responses:**   - No reply [1–4]: . |
| Other methods | None to report. |
| **Search strategies** | |
| Full search strategies | **Date search performed: 13^th^ of August 2024**  PubMed (6,843 results):   1. ((((((Myocardial Infarction OR MI OR “heart attack” OR “Cardiac Infarction” OR AMI OR “Acute Coronary Syndrome” OR ACS)   AND   1. (medic* OR drug* OR treatment* OR therap* OR prescription*))   AND   1. (Adher* OR Complian* OR Concordance OR tak* OR manag*))   AND   1. (Barrier* OR facilitator* OR enabl* OR limit* OR prevent* OR factor* OR determinan* OR predict* OR variable* OR challeng* OR implement* OR issue* OR Influen* OR problem* OR reason*))   AND   1. (home OR Domiciliary OR domestic OR Primary Care OR Community OR ambulatory OR Discharge OR cardiac Rehab*))   AND   1. (Patient* OR Family Member OR Care* OR Spous* OR advocate* OR daughter* OR son* OR sister* OR brother* OR father* OR mother* OR relative*))   AND   1. (Behavior* OR Behaviour* OR Attitude* OR view* OR Belief* OR Knowledge OR Perspective* OR understand* OR Opinion* OR Experience*)   SCOPUS (2,158 results), :   1. ( TITLE-ABS-KEY ( "myocardial infarction" OR mi OR "heart attack" OR "cardiac infarction" OR AMI OR "acute coronary syndrome" OR ACS ) )   AND   1. ( TITLE-ABS-KEY ( medic* OR drug* OR treatment* OR therap* OR prescription* ) )   AND   1. ( TITLE-ABS-KEY ( adher* OR complian* OR concordance OR tak* OR manag* ) )   AND   1. ( TITLE-ABS-KEY ( barrier* OR facilitator* OR enabl* OR limit* OR prevent* OR factor* OR determinan* OR predict* OR variable* OR challeng* OR implement* OR issue* OR influen* OR problem* OR reason* ) )   AND   1. ( TITLE-ABS-KEY ( home OR domiciliary OR domestic OR "primary care" OR community OR ambulatory OR discharge OR "cardiac rehab*" ) )   AND   1. ( TITLE-ABS-KEY ( patient* OR "family member" OR care* OR spous* OR advocate* OR daughter* OR son* OR sister* OR brother* OR father* OR mother* OR relative* ) )   AND   1. ( TITLE-ABS-KEY ( behavior* OR behaviour* OR attitude* OR view* OR belief* OR knowledge OR perspective* OR understand* OR opinion* OR experience* ) )   Embase using Emtree (2,146 results):  #1 ('Myocardial Infarction' OR MI OR 'heart attack' OR 'Cardiac Infarction' OR AMI OR 'Acute Coronary Syndrome' OR ACS)  #2 ( medic* OR drug* OR treatment* OR therap* OR prescription*)  #3 (Adher* OR Complian* OR Concordance OR tak* OR manag*)  #4 (Barrier* OR facilitator* OR enabl* OR limit* OR prevent* OR factor* OR determinan* OR predict* OR variable* OR challeng* OR implement* OR issue* OR Influen* OR problem* OR reason*)  #5 ( home OR Domiciliary OR domestic OR 'Primary Care' OR Community OR ambulatory OR Discharge OR 'cardiac Rehab*')  #6 (Patient* OR Family Member OR Care* OR Spous* OR advocate* OR daughter* OR son* OR sister* OR brother* OR father* OR mother* OR relative*)  #7 (Behavior* OR Behaviour* OR Attitude* OR view* OR Belief* OR Knowledge OR Perspective* OR understand* OR Opinion* OR Experience*)  **#1 AND #2 AND #3 AND #4 AND #5 AND #6 AND #7**  CINAHL (1,034 results):   1. Myocardial Infarction OR MI OR “heart attack” OR “Cardiac Infarction” OR AMI OR “Acute Coronary Syndrome” OR ACS   AND   1. medic* OR drug* OR treatment* OR therap* OR prescription*   AND   1. Adher* OR Complian* OR Concordance OR tak* OR manag*   AND   1. Barrier* OR facilitator* OR enabl* OR limit* OR prevent* OR factor* OR determinan* OR predict* OR variable* OR challeng* OR implement* OR issue* OR Influen* OR problem* OR reason*   AND   1. home OR Domiciliary OR domestic OR Primary Care OR Community OR ambulatory OR Discharge OR cardiac Rehab*   AND   1. Patient* OR Family Member OR Care* OR Spous* OR advocate* OR daughter* OR son* OR sister* OR brother* OR father* OR mother* OR relative*   AND   1. Behavior* OR Behaviour* OR Attitude* OR view* OR Belief* OR Knowledge OR Perspective* OR understand* OR Opinion* OR Experience*   **Date search performed: 13^th^ of August 2024:**  Web of Science (1,822 results):   1. Topic: “Myocardial Infarction” OR MI OR “heart attack” OR “Cardiac Infarction” OR AMI OR “Acute Coronary Syndrome” OR ACS   AND   1. Topic: medic* OR drug* OR treatment* OR therap* OR prescription*   AND   1. Topic: Adher* OR Complian* OR Concordance OR tak* OR manag*   AND   1. Topic: Barrier* OR facilitator* OR enabl* OR limit* OR prevent* OR factor* OR determinan* OR predict* OR variable* OR challeng* OR implement* OR issue* OR Influen* OR problem* OR reason*   AND   1. Topic: home OR Domiciliary OR domestic OR “Primary Care” OR Community OR ambulatory OR Discharge OR “cardiac Rehab*”   AND   1. Topic: Patient* OR “Family Member” OR Care* OR Spous* OR advocate* OR daughter* OR son* OR sister* OR brother* OR father* OR mother* OR relative*   AND   1. Topic: Behavior* OR Behaviour* OR Attitude* OR view* OR Belief* OR Knowledge OR Perspective* OR understand* OR Opinion* OR Experience* |
| Limits and restrictions | None used. |
| Search filters | In SCOPUS data base a search filter was used excluding Engineering and CS. This reduced the search results from 2009-1975 |
| Prior work | The search strategy of a previous systematic review was viewed when designing the above search strategies of this systematic review, to help with selecting synonyms for terms to optimise the number of records retrieved [5]. |
| Updates | Searches were repeated on the 13/08/2024 to capture further relevant studies published since the first searches. No further relevant studies were captured in the updated search. |
| Dates of searches | A comprehensive literature search was initially run on April-June 2023 and then re-run 13 of August 2024. |
| **Peer review** | |
| Peer review | The first author initially developed the search strategy with the help of Ms Virginia Conrick, Academic Success Librarian, Boole Library, University College Cork, via online Microsoft^®^ Teams meeting and then peer-reviewed as a live document on Microsoft^®^ Teams by two supervisors. Revisions were proposed in an iterative review process of the search strategy, discussed between the supervisors and the first author, and implemented as required. |
| **Managing records** | |
| Total Records | A total of 14,002 citations were retrieved from five databases searched till 13^th^ of August 2024 . See PRISMA Flow Diagram (Figure 1). |
| Deduplication | Duplicates were removed by the first author, using Zotero’s duplicate identification feature, and then records were screened manually by individually reviewing all remaining records to ensure all duplicates were removed. |

References

1. Bubnova MG, Aronov DM, Krasnitsky VB, et al. A home exercise training program after acute coronary syndrome and/or endovascular coronary intervention: Efficiency and a patient motivation problem. Ter Arkh [Internet]. 2014;86:23–32. Available from: https://www.scopus.com/inward/record.uri?eid=2-s2.0-84899155111&partnerID=40&md5=0eccbbb107b9beea2e40aa1db8c3d44d

2. Patton AP, Liu Y, Hartwig DM, et al. Community pharmacy transition of care services and rural hospital readmissions: A case study. J Am Pharm Assoc [Internet]. 2017;57:S252-S258.e3. Available from: https://www.embase.com/search/results?subaction=viewrecord&id=L616159608&from=export

3. Lim W-H, Chae I-H, Yoon C-H, et al. Comparison of dual antiplatelet therapy prescribed as one-pill versus two-pill regimen: A pooled analysis of individual patient data from the three MR-CAPCIS trials. Thromb Haemost [Internet]. 2016;116:78–86. Available from: https://www.embase.com/search/results?subaction=viewrecord&id=L611064015&from=export

4. O’Farrell P, Murray J, Hotz S. Psychologic distress among spouses of patients undergoing cardiac rehabilitation. HEART & LUNG. 2000;29:97–104.

5. Fuller RH, Perel P, Navarro-Ruan T, et al. Improving medication adherence in patients with cardiovascular disease: a systematic review. Heart. 2018;104:1238–43.
